# Sugar in the Fluid of Ascites in a Diabetic Patient

**Published:** 1847-04

**Authors:** 


*Sugar in the fluid of Ascites in a Diabetic Patient.*.—In the fluid from the abdomen of a diabetic patient who was attacked with ascites, Dr. Landerer detected evident traces of the presence of sugar. The existence of this substance is almost all the secreted fluids in the body of persons affected with diabetes has now been proved.—*Heller's Archiv.*

